# Microvascular Changes after Epiretinal Membrane Vitrectomy with Intraoperative Intravitreal Dexamethasone Implant: An OCT Angiography Analysis

**DOI:** 10.3390/diagnostics14040411

**Published:** 2024-02-13

**Authors:** Antonio Baldascino, Matteo Mario Carlà, Lorenzo Vielmo, Gloria Gambini, Francesca Carolina Marzano, Fabio Margollicci, Nicola Claudio D’Onofrio, Stanislao Rizzo

**Affiliations:** 1 Ophthalmology Department, Fondazione Policlinico Universitario A. Gemelli, IRCCS, 00168 Rome, Italy; antonio.baldascino@policlinicogemelli.it (A.B.); gambini.gloria@gmail.com (G.G.); liccifabio@gmail.com (F.M.); nicola.c.donofrio@gmail.com (N.C.D.); stanislao.rizzo@gmail.com (S.R.); 2 Ophthalmology Unit, Catholic University “Sacro Cuore”, 00168 Rome, Italy; 3 Faculty of Medicine and Surgery, Catholic University “Sacro Cuore”, 00168 Rome, Italy; f.carolinamarzano@gmail.com

**Keywords:** epiretinal membrane, optical coherence tomography angiography, inflammation, intravitreal dexamethasone, Ozurdex, retinal plexi

## Abstract

Background: We aimed to explore microvascular changes evaluated with optical coherence tomography angiography (OCTA) in patients undergoing epiretinal membrane (ERM) pars-plana vitrectomy (PPV) combined with intravitreal Ozurdex implantation, compared with standard PPV. (2) Methods: Prospective interventional analysis on 25 eyes undergoing PPV + Ozurdex (Group A) and 25 eyes undergoing PPV alone. Best corrected visual acuity (BCVA) and OCTA parameters, such as vessel density (VD) of the superficial and deep capillary plexi (SCP and DCP) in the whole 6.4 mm × 6.4 mm and fovea area, were evaluated preoperatively and 3 months after surgery. (3) Results: Postoperative BCVA significantly improved in both groups. No cases of post-operative cystoid macular edema (CME) were reported in Group A vs. two eyes in Group B. In Group A we found a statistically significant increase of SCP’s VD in either the whole image (from 42.1 ± 4.1 to 45.6 ± 4.3%, *p* = 0.01) and the fovea image (from 38.5 ± 7.5 to 41.7 ± 4.2%, *p* = 0.03). In Group B, we reported no significant variations in the SCP’s VDs. In the DCP, VD significantly increased only in the whole image in Group A. Stage 4 ERMs showed the greatest improvement in VD, especially in Group A. (4) Conclusions: Intraoperative Ozurdex prompted a significant BCVA recovery and limited the occurrence of postoperative CME compared to the standard procedure. Moreover, Ozurdex implant is associated with a better restoration of microvascular structure in SCP and DCP.

## 1. Introduction

Idiopathic epiretinal membrane (iERM) is a frequently encountered vitreoretinal disorder affecting the macula, with an age-related increasing prevalence [1,2]. The advent of high-resolution imaging techniques, such as spectral domain- and swept source-optical coherence tomography (SD-OCT and SS-OCT), has significantly enhanced the understanding and evaluation of this condition [3,4]. Fundus oculi examination in patients with ERMs reveals evident vascular tortuosity and retinal contraction, although the quantification of these changes poses challenges. The introduction of optical coherence tomography angiography (OCTA), which is able to evaluate the tangential traction while taking into account the depth of the retinal distortions caused by the epiretinal traction via the inner and outer retinal layers, made it possible to measure various parameters, thanks to the en face visualization of the retinal plexuses, assessing the extent of distortion and the impact of ERM contractions on the retinal microvasculature [5,6].

The standard surgical approach for ERMs is vitrectomy combined with membrane peeling [7]. Nevertheless, the development of postoperative cystoid macular edema (CME) is a well-known side effect, hindering the possibility to achieve optimal visual outcomes [8,9]. To prevent CME development, the use of intraoperative intravitreal dexamethasone implant (Ozurdex^®^, Allergan Inc., Irvine, CA, USA) was proposed [10,11]. A recent meta-analysis compared ERM vitrectomy without and with intraoperative intravitreal Ozurdex, showing that the latter allowed for a better visual outcome at three months [12].

The evolution of vascular tortuosity after surgical ERM treatment has been studied with OCTA, showing that the reconstitution on vessel linearity around the macula was associated with better visual outcomes [13,14]. Similarly, Bacherini et al. showed significant changes in all retinal plexuses (superficial, deep and choriocapillaris), evolving in the six months after ERM surgery [15].

As far as we know, the application of OCTA in ERM vitrectomy combined with intraoperative dexamethasone implant, has not been explored yet. This paper aims to explore microvascular changes in patients undergoing Ozurdex-augmented ERM surgery, compared with standard surgical approach. Additionally, we investigated the anti-inflammatory and anti-angiogenic properties of dexamethasone to prevent postoperative CME.

## 2. Materials and Methods

In this prospective mono-centric randomized comparative analysis, we enrolled 50 eyes of 50 patients affected by iERMs at Policlinico Universitario Agostino Gemelli between 27 October 2021 and 20 June 2023. The Policlinico Universitario Agostino Gemelli Institutional Ethics Committee approved the study, which followed the tenets of the Declaration of Helsinki. All patients who were recruited provided signed, fully informed consent.

The inclusion criteria for patient selection were as follows: age > 18 years; clinical and instrumental diagnosis of primary epiretinal membrane (ERM) at stage 3–4 according to the OCT staging system proposed by Govetto et al. [16], and preoperative pseudophakia.

Exclusion criteria included patients with any kind of secondary ERM, concomitant ocular or systemic diseases that could affect best-corrected visual acuity (BCVA) and/or central macular thickness (CMT), degenerative myopia, ambient opacities hindering imaging and history of ocular surgeries, except for uncomplicated cataract surgery.

We divided our sample into two subgroups:

- Group A: eyes undergoing pars plana vitrectomy (PPV) combined with intraoperative intravitreal administration of dexamethasone implant (Ozurdex);
- Group B: eyes undergoing PPV and peeling without concomitant intravitreal corticosteroid administration.

After inclusion into the study, the participants were randomly assigned 1:1 to either Group A or Group B using a computer-generated randomization list.

Before the intervention, the participants’ eyes underwent a complete ophtalmological examination, with BCVA measurement, tonometry, slit-lamp biomicroscopy and fundus examination. OCT and OCT-A scans were performed under pharmacologically induced mydriasis with 1% tropicamide solution. These procedures were repeated at the follow-up visit 3 months after surgery. This timing was chosen based on the duration of dexamethasone implant’s therapeutic effect, as reported in the literature [17].

The main outcome of this research was to outline the changes in retinal plexuses after iERM surgery in the combined PPV-Ozurdex group compared to the PPV alone group. As secondary outcomes, we assessed changes in CMT, BCVA and the rate of postoperative CME between the two groups.

### 2.1. Scan Protocol

The OCT/OCT-A scans were performed using the full-range OCT Solix system (Optovue Inc., Freemont, CA, USA). Scans with low-quality indices (<7/10) due to significant lens opacities, frequent eyelid blinking, excessive motion artifacts, and other factors were rejected. The OCTA images were acquired using the AngioVue Retina software (Optovue Inc., Freemont, CA, USA), which automatically assessed the vascular density (VD) of the superficial and deep capillary plexuses (SCP, DCP) in the entire macular region corresponding to a 6.4 mm × 6.4 mm field centered on the fovea. Parameters such as Whole Image (the entire image) and Fovea (the area within the 1 mm central ring of the ETDRS grid) were evaluated. Additionally, the RetinaCube software evaluated the central macular thickness (CMT). For the SCP, en face images were obtained using the automatic segmentation between the inner boundary (internal limiting membrane, ILM) and the outer boundary (inner plexiform layer). The DCP was identified between the outer plexiform layer and the inner plexiform layer. Each OCTA image underwent motion correction and 3D projection artifact removal technology to improve image quality.

### 2.2. Surgical Procedure

Under peribulbar anesthesia, a standard 25-gauge 3-port pars plana vitrectomy was performed on each eye utilizing the Constellation Vision System (Alcon Laboratories Inc., Fort Worth, TX, USA) and a wide-angle noncontact viewing system (Resight^®^; Carl Zeiss Meditec AG, Jena, Germany). The ILM/ERM complex was dyed with MembraneBlue Dual, (TrypanBlue 0.15% + Brillian Blue G 0.025%, DORC, Zuidland, The Netherlands) and peeled using vitreoretinal forceps until reaching an area two papillary diameters away from the fovea. For eyes with retinal tears or holes, peripheral retinal photocoagulation was performed in addition to a full vitrectomy.

At the end of the surgery, in the first subgroup, following fluid–air exchange, the Ozurdex implant was injected through the inferotemporal sclerotomy after infusion cannula removal and repositioning in the supertemporal sclerotomy. The implant was always visualized in the vitreous cavity thanks to the wide angle viewing system.

In the non-Ozurdex subgroup, a fluid–air exchange was performed to terminate the surgery.

All surgeries were performed by two experienced surgeons (A.B. and S.R.).

### 2.3. Statistical Analysis

The sample size of each considered group was evaluated using the G-Power software package (Version 3.1.9.6). Assuming a minimum difference of 15%, a residual standard deviation of 10%, a power of 0.08 and an alpha of 0.05 to highlight the differences, the required smallest population size was 25 patients for each group.

Statistical analysis was conducted using GraphPad PRISM Software (Version 9.5; GraphPad, La Jolla, CA, USA). The Shapiro–Wilk test was employed to assess the normality of the sample, with a *p*-value greater than 0.05 supporting the null hypothesis of normal distribution. Mean values detected in the two groups at baseline were compared by a *t*-test. For the comparison of continuous variables between baseline and postoperative data, a Mann–Whitney U test was utilized with a 95% confidence interval (CI). ANOVA for repeated measures, treating Ozurdex implantation as covariate, was used to assess difference in vascular changes between groups. To explore the relationship between quantitative OCTA values and clinical outcomes (BCVA and CMT), a Spearman correlation was performed. Mean standard deviation (SD) was used to present quantitative data, and a significance level of *p* < 0.05 was employed to determine statistical significance.

## 3. Results

Demographic characteristics of the two subgroups are visible in Table 1. No significant differences were reported in preoperative data.

Mean preoperative BCVA was 0.41 ± 0.3 and 0.38 ± 0.3 Snellen equivalent in Group A and group B, respectively, improving significantly to 0.79 ± 0.2 and 0.71 ± 0.3 Snellen equivalent at 3 months (*p* = 0.008 and *p* = 0.001 for Group A and B, respectively). The difference in terms of visual acuity was not significant between the two groups (*p* = 0.054). Mean postoperative IOP showed mild but not significant elevation in Group A (from 14.1 ± 2.9 to 17.6 ± 3.1, range 13–22 mmHg, *p* = 0.11), while remaining stable in Group B (from 15.0 ± 4.3 to 15.8 ± 5.1, *p* = 0.88). One patient had mild ocular hypertension (IOP 22 mmHg) at 3 months, but it was fully resolved without medical treatment. None of the patients required IOP-lowering treatment.

Mean CMT at baseline was 485 ± 63 µm and 467 ± 93 µm in Group A and B, respectively. At 3 months, a significative improvement was seen in Group A (mean CMT 358 ± 62 µm, *p* = 0.001) and Group B (mean CMT 377 ± 53 µm, *p* = 0.009).

There were no complications related to the Ozurdex implant injection procedure in an air-filled eye at the end of the surgery. After the injection, the implant was visible on the inferior retina with no apparent hemorrhage or trauma.

In Group A, we reported no cases of post-operative CME, while in Group B, 2 eyes had CME at the 3 months follow-up’s OCT scans.

### 3.1. OCTA Findings

Concerning the SCP, in Group A we found a statistically significant increase of VD in either the whole image (42.1 ± 4.1 vs. 45.6 ± 4.3% from baseline to the 3 months follow-up, *p* = 0.01) and the fovea image (38.5 ± 7.5 vs. 41.7 ± 4.2% from baseline to the 3 months follow-up, *p* = 0.03). In Group B, we reported no significant variations in the VD of the whole area (42.9 ± 5.3% at baseline vs. 43.8 ± 5.8% at the 3 months follow-up, *p* = 0.69) and the fovea area (40.4 ± 6.7 vs. 40.9 ± 6.7% from baseline to the 3 months follow-up, *p* = 0.25). The differences at three months between Group A and B changes were significant for the whole image’s VD (*p* = 0.01), but not significant for the fovea image (*p* = 0.49).

By measuring the VD of the DCP before and after surgery, in Group A we observed a significant improvement in the whole image (from 40.4 ± 10.1% at baseline to 44.7 ± 6.6% at the 3 months follow-up, *p* = 0.005), but not in the fovea area (30.7 ± 10.0 vs. 32.3 ± 10.9% from baseline to the 3 months follow-up, *p* = 0.14). In Group B, we reported no significant changes in both areas: 41.8 ± 13.2 vs. 42.0 ± 11.5% in the whole image from baseline to the 3 months follow-up, *p* = 0.26; 30.0 ± 7.7 vs. 31.0 ± 8.9% in the fovea image from baseline to the 3 months follow-up, *p* = 0.11. Overall, the changes in DCP VD differed between the two groups in the whole area (*p* = 0.016), but not in the fovea area (*p* = 0.38).

**Figure 1:**
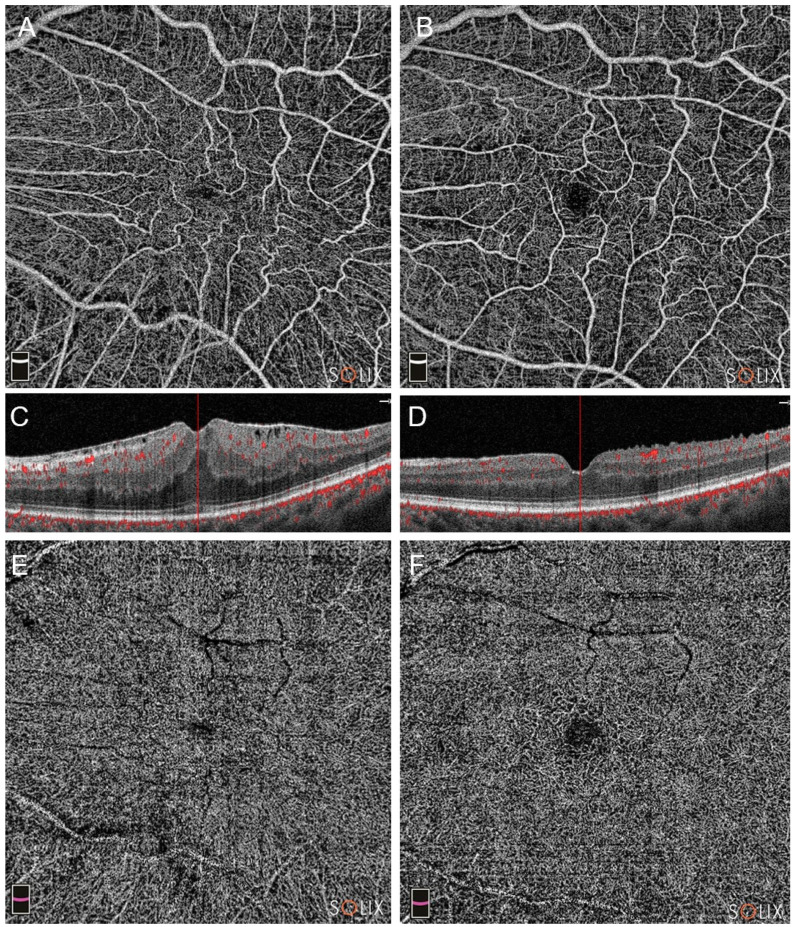
Optical coherence tomography analysis (OCTA) of a patient in Group A (pars plana vitrectomy with intraoperative Ozurdex implantation), affected by a stage 3 epiretinal membrane (ERM). Preoperative (**A**,**C**,**E**) and postoperative scans at three months (**B**,**D**,**F**) are shown. Note the improvement in vascular network in either the superficial capillary plexus (SCP, images (**A**,**B**)) and the deep capillary plexus (DCP, (**E**,**F**)). The B-scans highlight a significant improvement, too (scans (**C**,**D**)).

**Figure 2:**
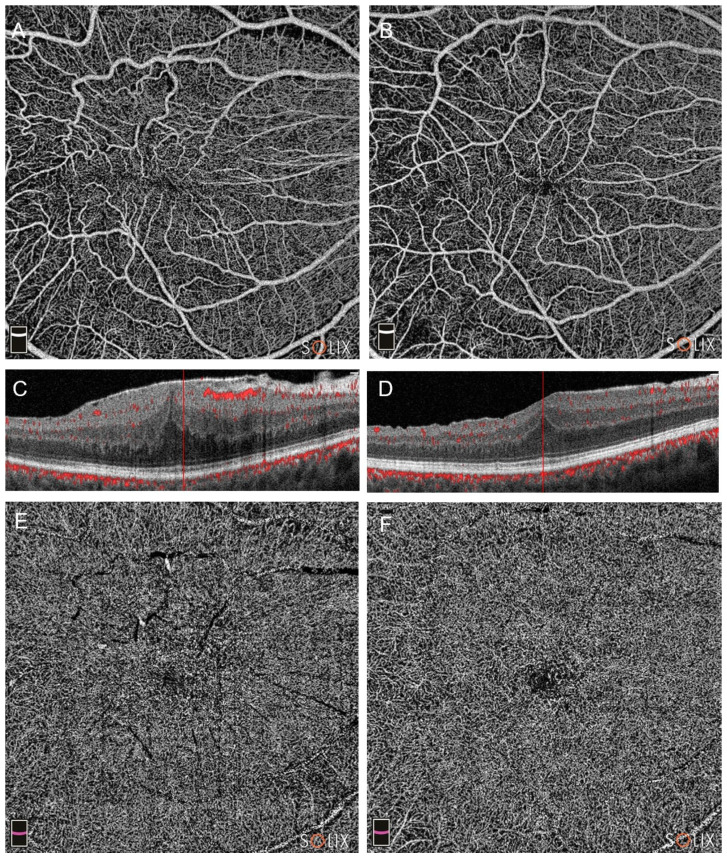
Optical coherence tomography analysis (OCTA) of a patient in Group B (pars plana vitrectomy alone), affected by a stage 3 epiretinal membrane (ERM). Preoperative (**A**,**C**,**E**) and postoperative scans at three months (**B**,**D**,**F**) are shown, with the evolution of the superficial capillary plexus (SCP, images (**A**,**B**)), the deep capillary plexus (DCP, (**E**,**F**)) and the B-scans (**C**,**D**).

Pre and post-operative OCTA of Group A and Group B patients are visible in Figure 1 and Figure 2.

Table 2 summarizes the differences between the two groups at 3 months.

### 3.2. Correlation Analysis

Before surgery, negative correlations were found between the Govetto stages and VD of the SCP and the DCP whole area (r = −0.2108, *p* = 0.04 for the SCP; r = −0.3591, *p* = 0.02 for the DCP) of the entire cohort. Similar results were found for the fovea area (r = −0.3045 and r = −0.3869 for SCP and DCP, respectively) of the entire cohort.

Postoperative analysis in patients undergoing PPV + Ozurdex (Group A), highlighted no correlation between variations in SCP whole area’s VD and CMT (r: −0.1756, *p*, 0.29), neither when correlating variations in DCP whole area’s VD and CMT (r: 0.1137, *p*, 0.47). Conversely, SCP and DCP variations positively correlated with BCVA improvement, and this correlation was significant for the DCP (r: 0.3610, *p*, 0.08 for SCP vs. BCVA; r: 0.4520, *p*, 0.03 for DCP vs. BCVA).

### 3.3. Stage 3 vs. Stage 4

Successively, we performed a subgroup analysis based on the stage of the epiretinal membrane. The complete comparison is summarized in Table 3.

**Figure 3:**
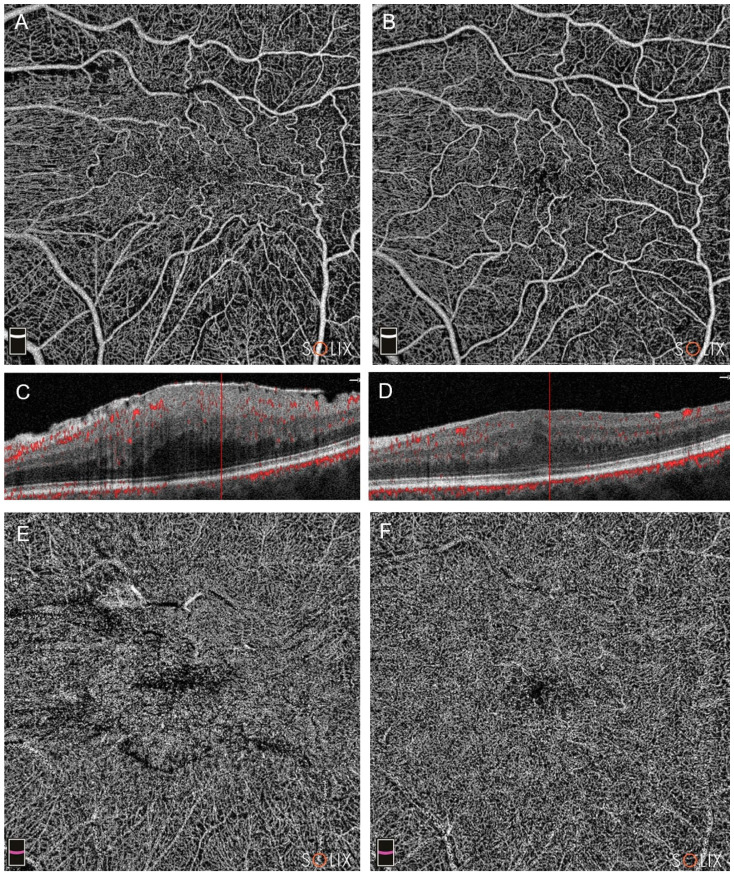
Optical coherence tomography analysis (OCTA) of a patient in Group A (pars plana vitrectomy with intraoperative Ozurdex implantation), affected by a stage 4 epiretinal membrane (ERM), showing preoperative (**A**,**C**,**E**) and postoperative scans at three months (**B**,**D**,**F**). In patients with stage 4, a significant microvascular restoration was reported, in either the superficial capillary plexus (SCP, images (**A**,**B**)) and the deep capillary plexus (DCP, (**E**,**F**)). Note the partial restoration of retinal layers in B-scans (scans (**C**,**D**)).

In stage 4 ERMs, we reported significant changes in all parameters in Group A (all *p*-values < 0.05) (Figure 3).

**Figure 4:**
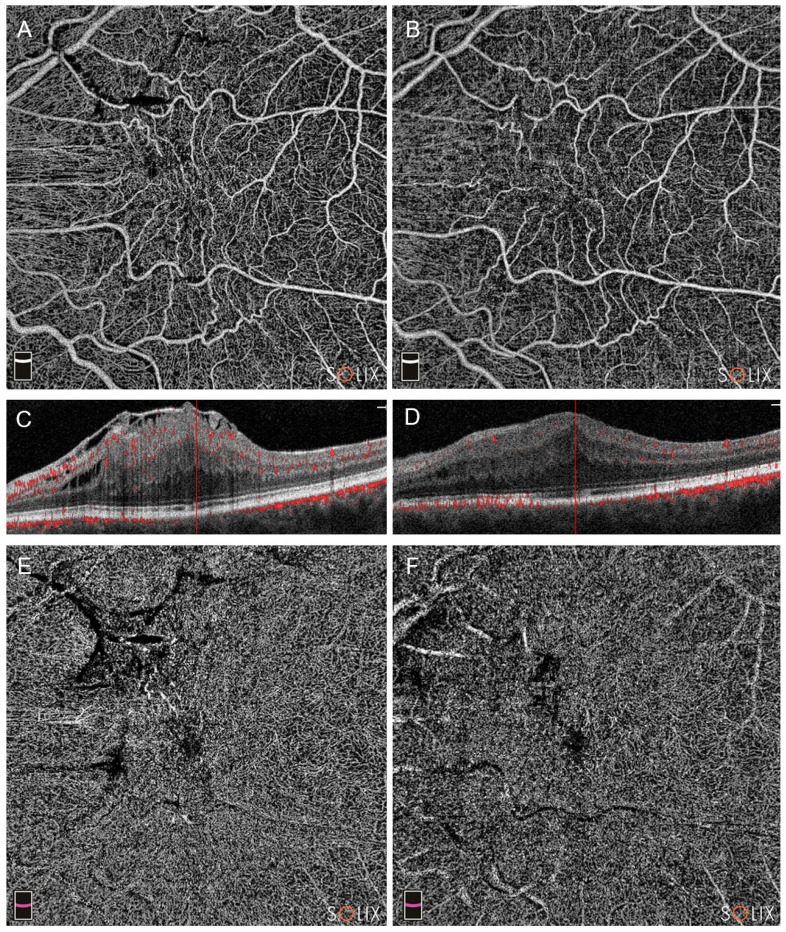
Optical coherence tomography analysis (OCTA) of a patient in Group B (pars plana vitrectomy alone), affected by a stage 4 epiretinal membrane (ERM), showing preoperative (**A**,**C**,**E**) and postoperative scans at three months (**B**,**D**,**F**). Note the changes in superficial capillary plexus (SCP, images (**A**,**B**)), deep capillary plexus (DCP, (**E**,**F**)) and the partial restoration of retinal layers in B-scans (scans (**C**,**D**)).

Conversely, in Group B we found significant changes only in the VD of the SCP in the whole the fovea areas (Figure 4).

In stage 3 ERMs, Group B did not show significant changes in any of the studied parameters at 3 months (all *p* > 0.05). Meantime, Group A showed a significant increase in whole images in both SCP and DCP’s VD.

## 4. Discussion

With the advent of OCTA, retinal microvasculature has been a subject of research in various retinal disorders, with several works analyzing microvascular changes caused by ERM and their evolution after surgical peeling [6,15]. In this study, we aimed to analyze the effects of an intravitreal dexamethasone implant (Ozurdex), in the context of ERM surgery, on macular microvascular homeostasis. In the PPV + Ozurdex group, we highlighted the significant increase in vessel densities of the SCP and DCP at the 3 months follow-up, suggesting significant postoperative microvascular remodeling. Conversely, no significant changes in terms of vessel density were found in the PPV alone group.

In vitreoretinal interface disorders, such as ERMs, it is hypothesized that the macular distortion caused by the contractile membrane may initiate the inflammatory cascade [18]. Within this context, the utilization of the intravitreal Ozurdex implant has been investigated, since dexamethasone (DEX) is recognized for its potent anti-inflammatory properties and favorable safety profile [19]. The intravitreal dexamethasone implant is authorized for use in Europe for the management of posterior segment inflammation resulting from noninfectious uveitis, CME associated with retinal vein occlusion, and diabetic macular edema (DME) [11]. Furthermore, the use of dexamethasone has been documented in various other inflammatory conditions, including pseudophakic CME, [10] inflammation following retinal detachment ab externo repair surgery, [20] and exacerbation of macular edema following cataract surgery [21].

One notable benefit of this sustained-release implant is its effectiveness in vitrectomized eyes, which may not be as responsive to intravitreal anti-vascular endothelial growth factor (anti-VEGF) therapy due to a more rapid clearance or washout effect [22]. Its use has been studied in the last few years in patients for ERM removal and showed promising results, both anatomically and functionally [12,23,24,25,26]. In particular, Iovino et al. and Fallico et al. demonstrated how eyes undergoing ERM vitrectomy combined with intraoperative dexamethasone implantation has better visual outcomes at a 15-day follow-up and 3-month follow-up, compared to those undergoing ERM vitrectomy without dexamethasone implantation, with a significant improvement in macular thickness in the immediate postoperative period correlating with better visual recovery [25,27]. Similarly, we reported better BCVA at three months in the PPV + Ozurdex group, even though more research is needed to prove the results to be significant.

Studies on OCTA showed that, in case of ERM development, SCP is significantly impaired, probably due to the ERM-associated direct mechanical stress affecting the inner retinal layer and causing progressive capillary subocclusion [28]. Lin et al. demonstrated that the tractional force of ERM affected not only the tortuous SCP, but also the DCP, which presented focal areas of non-perfusion, and their results were confirmed by successive studies [29,30]. Mastropasqua et al. showed that the perfusion density (PD) and VD were statistically lower in ERM-affected eyes than that in the control group [31]. Furthermore, according to Rommel et al., ERMs influence choroidal and choriocapillary networks, and damage in the DCP and SCP may therefore have an impact on the choriocapillaris’ microvasculature [32].

In a 6-month study of ERMs undergoing PPV and peeling, Bacherini et al. observed a considerable rise in SCP’s VD, likely due to its increased sensitivity to the reopening of small vessels that were suboccluded during the preoperative period. Moreover, the DCP showed a marked increase in VD and PD during the follow-ups. The removal of the ERM seemed to induce a gradual and stable reopening of retinal vessels, which were emptied by tractional stretching forces, but vascular walls were conceptually unaltered. Therefore, the postoperative increase in blood flow may last for several months [15]. Interestingly, in the first month after surgery, they reported a decrease in SCP and DCP’s VDs [15]. Li et al. previously reported no changes in SCP and DCP 1 month after surgery, but reported an increased arterial oxygen saturation. They hypothesized that the removal of the vitreous body determines an improvement in retinal blood flow. As a consequence, there is a gradual increase in retinal arteriolar saturation, with better oxygen diffusion and transport from the anterior segment. Conversely, there was no corresponding change in venous saturation, suggesting an improved utilization of oxygen [33].

In our research, we reported an improvement in vascular parameters in the PPV + Ozurdex group, rather than the PPV alone group. Previous studies showed that Ozurdex determines a leukostasis inhibition, a VEGF reduction and a tightening of the vascular endothelial tight junctions with reduced capillary leakage, as well as a block of the inflammatory cascade [34,35,36]. In an OCTA analysis, Minnella et al. highlighted significant microvascular remodeling after an intravitreal dexamethasone implant in both DME and retinal vein occlusion (RVO). In particular, they reported vascular restoration mainly concerning the DCP in the DME group and both the SCP and DCP in eyes affected by RVO. Even though ERM-induced CME and microvascular changes do not rely on a capillary leakage mechanism, we hypothesize that dexamethasone implant, in addition to reducing the rate of post-vitrectomy CME (no cases in PPV + Ozurdex group vs. two cases in the PPV alone group), may allow for faster microvascular restoration in either the SCP and DCP. We indeed reported higher SCP and DCP’s VD in the PPV + Ozurdex group, compared to the PPV alone group, and those values, specifically the DCP’s VD raise, positively correlated with postoperative BCVA improvement.

Consistently with the study of Bacherini et al., we found a negative correlation between SCP and DCP’s VD and the stage of the pathology, following Govetto’s classification [15,16]. Moreover, stage 4 ERMs, starting from lower VD values, showed a more significant microvascular improvement, suggesting an even more pronounced postoperative remodeling in these advanced conditions. Nevertheless, postoperative VD values remained in all instances lower than those of stage 3 ERMs. Finally, SCP improvements in either stage 3 (2.8%) and stage 4 (3.9%) ERMs are higher than the coefficient of repeatability (CoR) recently claimed for parafoveal VD, [37] suggesting that these differences indicate a true-to-life improvement in macular microvascular architecture.

Our study has several limitations; firstly, the small sample size may have impacted statistical significance. However, this result represents an important starting point for expanding the study to a larger cohort of patients with the aim of using intravitreal Ozurdex implantation as a standard intraoperative protocol. Secondly, the short follow-up period may have hampered the possibility of evaluating long term microvascular changes. Nevertheless, we consciously decided to evaluate three-month results to assess the effects of a dexamethasone implant at the end of its period of action.

## 5. Conclusions

In conclusion, we can affirm that intraoperative intravitreal Ozurdex administration is associated with good functional and anatomical recovery after ERM surgery, limiting the occurrence of postoperative CME compared to the standard procedure. Furthermore, we hypothesize that the blockage of the cytokine cascade and the tight junction tightening are at the basis of the microvascular restoration we reported after Ozurdex implantation. Further studies with a larger study population and longer follow-ups are needed to corroborate these findings.

## Figures and Tables

**Table 1:** Baseline demographic and clinical characteristics of the study population.

Characteristics (Mean ± SD)	PPV + Ozurdex (*n* = 25)	PPV Alone (*n* = 25)	*p*
Study eye, RE, no (%)	12 (48%)	13 (52%)	0.81
Age, years	68.2 ± 4.8	69.7 ± 5.3	0.57
Gender, male, no (%)	11 (44%)	14 (56%)	0.49
Preoperative BCVA, decimals	0.41 ± 0.3	0.38 ± 0.3	0.53
Preoperative IOP, mmHg	14.1 ± 2.9	15.0 ± 4.3	0.64
Stage 3/4 (%/%)	14/11 (56%/44%)	12/13 (48%/52%)	0.18
OCT/OCTA features			
CMT, µm	485 ± 63	467 ± 93	0.21
SCP whole VD, %	42.1 ± 4.1	42.9 ± 5.3	0.57
SCP fovea VD, %	38.5 ± 7.5	40.4 ± 6.7	0.09
DCP whole VD, %	40.4 ± 10.1	41.8 ± 13.2	0.23
DCP fovea VD, %	30.7 ± 10.0	30.0 ± 7.7	0.14

SD = standard deviation; RE = right eye; PPV = pars plana vitrectomy; BCVA = Best Corrected Visual Acuity; IOP = intraocular pressure; OCT = optical coherence tomography; OCTA = optical coherence tomography angiography; CMT = central macular thickness; SCP = superficial capillary plexus; DCP = deep capillary plexus; VD = vessel density.

**Table 2:** OCTA parameters at the 3 months follow-up, compared between Group A (PPV + Ozurdex) and Group B (PPV alone).

OCTA Parameters at 3 Months (Mean ± SD)	PPV + Ozurdex (*n* = 25)	PPV Alone (*n* = 25)	*p* ^Ϯ^
SCP whole VD, %	45.6 ± 4.3	43.8 ± 5.8	0.01 *
SCP fovea VD, %	41.7 ± 4.2	40.9 ± 6.7	0.49
DCP whole VD, %	44.7 ± 6.6	42.0 ± 11.5	0.016 *
DCP fovea VD, %	32.3 ± 10.9	31.0 ± 8.9	0.38

^Ϯ^ ANOVA for repeated measures. PPV = pars plana vitrectomy; OCTA = optical coherence tomography angiography; SCP = superficial capillary plexus; DCP = deep capillary plexus; VD = vessel density. * stands for statistical significance.

**Table 3:** OCTA characteristics of the PPV + Ozurderx and PPV alone subgroups, divided for ERM stage, according to Govetto et al. [16].

Characteristics (Mean ± SD)	PPV + Ozurdex (*n* = 25)		*p*	PPV Alone (*n* = 25)		*p*
Stage 3	Baseline	3 Months		Baseline	3 Months	
SCP whole VD, %	43.5 ± 6.1	46.3 ± 8.3	0.01 *	43.9 ± 5.2	44.7 ± 5.6	0.36
SCP fovea VD, %	40.5 ± 9.5	42.1 ± 8.4	0.10	41.3 ± 6.4	41.0 ± 6.2	0.64
DCP whole VD, %	41.3 ± 11.3	45.9 ± 7.8	0.04 *	42.4 ± 12.8	42.1 ± 11.5	0.44
DCP fovea VD, %	31.7 ± 10.3	32.6 ± 9.1	0.21	30.9 ± 11.7	31.5 ± 10.2	0.22
Stage 4						
SCP whole VD, %	40.8 ± 4.3	44.7 ± 5.3	0.003 *	41.3 ± 4.3	42.9 ± 4.8	0.03 *
SCP fovea VD, %	36.9 ± 6.1	40.4 ± 8.2	0.02 *	39.9 ± 7.0	40.8 ± 5.5	0.04 *
DCP whole VD, %	39.0 ± 8.9	43.7 ± 5.4	0.01 *	41.2 ± 11.2	41.9 ± 9.5	0.12
DCP fovea VD, %	29.8 ± 9.6	31.6 ± 6.5	0.03 *	29.3 ± 9.0	30.6 ± 7.2	0.08

PPV = pars plana vitrectomy; OCTA = optical coherence tomography angiography; ERM = epiretinal membrane; SCP = superficial capillary plexus; DCP = deep capillary plexus; VD = vessel density. Asterisks stand for statistical significance.

## Data Availability

The data that support the findings of this study are available from the corresponding author, M.M.C., upon reasonable request.
